# Assessing resting-state brain functional connectivity in adolescents and young adults with narcolepsy using functional near-infrared spectroscopy

**DOI:** 10.3389/fnhum.2024.1373043

**Published:** 2024-03-28

**Authors:** Chen Wenhong, Mo Xiaoying, Shi Lingli, Tang Binyun, Wen Yining, Zhao Mingming, Lu Yian, Qin Lixia, Hu Wenyu, Pan Fengjin

**Affiliations:** ^1^Department of Sleep Medicine, The People’s Hospital of Guangxi Zhuang Autonomous Region, Nanning, Guangxi, China; ^2^Guangxi Clinical Reserch Center for Sleep Medicine, The People's Hospital of Guangxi Zhuang Autonomous Region, Nanning, Guangxi, China

**Keywords:** narcolepsy, fNIRS, functional connectivity, graph theory, prefrontal cortex

## Abstract

This study aimed to elucidate the alterations in the prefrontal cortex’s functional connectivity and network topology in narcolepsy patients using functional near-infrared spectroscopy (fNIRS). Twelve narcolepsy-diagnosed patients from Guangxi Zhuang Autonomous Region’s People’s Hospital Sleep Medicine Department and 11 matched healthy controls underwent resting fNIRS scans. Functional connectivity and graph theory analyses were employed to assess the prefrontal cortex network’s properties and their correlation with clinical features. Results indicated increased functional connectivity in these adolescent and young adult patients with narcolepsy, with significant variations in metrics like average degree centrality and node efficiency, particularly in the left middle frontal gyrus. These alterations showed correlations with clinical symptoms, including depression and sleep efficiency. However, the significance of these findings was reduced post False Discovery Rate adjustment, suggesting a larger sample size is needed for validation. In conclusion, the study offers initial observations that alterations in the prefrontal cortex’s functional connectivity may potentially act as a neurobiological indicator of narcolepsy, warranting further investigation with a larger cohort to substantiate these findings and understand the underlying mechanisms.

## 1 Introduction

Narcolepsy is a chronic neurological disorder that affects approximately 1 in 2,000 individuals, characterized by excessive daytime sleepiness, cataplexy, sleep paralysis, and hallucinations (Silber et al., 2002; Dauvilliers et al., 2003). The exact cause of the disorder is still unknown; however, studies suggest it may be associated with the destruction of hypocretin (orexin)-producing neurons in the hypothalamus, which are integral to the regulation of sleep–wake cycles. The loss of these neurons disrupts rapid eye movement (REM) sleep regulation, leading to unstable transitions between wakefulness and REM sleep (Kroeger and de Lecea, 2009). These neurons have extensive projections to other brain regions, and their absence can disrupt the functioning of networks within the frontal lobe, limbic system, diencephalon, and brainstem (Peyron et al., 1998; Bassetti et al., 2019), contributing to the various symptoms observed in narcolepsy.

Neuroimaging studies have provided insights into the structural, metabolic, and functional connectivity changes associated with narcolepsy. For instance, a PET/CT study revealed that patients with type 1 narcolepsy exhibit pronounced hypometabolism in regions such as the middle frontal lobe and angular gyrus-areas linked to learning and memory functions-which correlates with persistent sleep/wake disturbances, increased Epworth Sleepiness Scale (ESS) scores, more frequent diurnal REM sleep disturbances, and nocturnal sleep fragmentation (Huang et al., 2018). Additionally, hippocampal and amygdala atrophy has been associated with increased daytime sleepiness and decreased REM sleep latency (Kim et al., 2016).

Beyond typical sleep-related symptoms, individuals with narcolepsy often experience cognitive deficits and mood disturbances, including depression and socialization challenges (Rieger et al., 2003; Naumann et al., 2006; Aran et al., 2010; Nevsimalova et al., 2011; Peraita-Adrados et al., 2011). A diffusion tensor imaging (DTI) study highlighted widespread impairment in the white matter integrity of the frontal lobe in drug-naïve patients with narcolepsy, which correlated with depressive symptomatology (Park et al., 2016). Huang et al. demonstrated that hypometabolism in the middle frontal lobe and angular gyrus is associated with somnolence and neurocognitive performance in young patients with narcolepsy (Huang et al., 2016).

The prefrontal cortex (PFC) is critical for attention, memory, and executive functions (Moraes et al., 2012), as well as being involved in daytime sleepiness (Killgore et al., 2012) and positive mood regulation (Oishi et al., 2013). And these are most likely related by lower hypothalamic secretin (Salomon et al., 2003; Brundin et al., 2007; Ponz et al., 2010; Ballotta et al., 2021). However, the functional connectivity patterns of the PFC in patients with narcolepsy have not been well characterized. Previous studies based on sMRI, fMRI, DTI, and PET/CT have yielded conflicting results (Dang-Vu, 2013; Cavaliere et al., 2020). A large number of structural nuclear magnetic resonance and functional magnetic resonance studies in resting state and task state have proved that the thickness and volume of prefrontal cortex decreased (Joo et al., 2009; Kim et al., 2009; Jeon et al., 2020), the intensity of functional connection decreased (Wu et al., 2022), and the activity of low frequency amplitude decreased (Fulong et al., 2018), and it was related to clinical features, including cognitive, memory and emotional abnormalities (Engström et al., 2014; Huang et al., 2016; Cavaliere et al., 2020), which was supported by the hypometabolism of prefrontal cortex shown by PET/CT (Huang et al., 2018). However, the research field is not always consistent. On the contrary, there are a series of research results that contradict the above findings. Specifically, a number of magnetic resonance imaging studies have shown that patients with narcolepsy have enhanced functional connections in the prefrontal cortex (Xiao et al., 2019; Fulong et al., 2020) and increased cortical thickness (Schaer et al., 2012), and PET/CT results also reveal the hypermetabolic state of the prefrontal cortex (Dauvilliers et al., 2010, 2017). These findings suggest that there may be a more complex regulatory mechanism for the role of prefrontal cortex in narcolepsy, which needs to be further explored and verified.

Near infrared spectroscopy (NIRS), like fMRI, measures hemodynamic changes related to neural activity, but uses near-infrared light instead of a magnetic field. Compared with magnetic resonance, it has the advantages of portable, low-cost, quiet, and unlimited, so it has attracted the attention of many researchers (Ferrari and Quaresima, 2012), Especially in schizophrenia (Sugimura et al., 2014; Yanagi and Shirakawa, 2021), depression (Chao et al., 2021), bipolar disorder (Ma et al., 2020), as well as Alzheimer’s disease (Li et al., 2018), Obstructive sleep apnea (Mingming et al., 2024), etc.

Narcolepsy often occurs in adolescents and young adults, significantly impacting their cognitive, emotional, and social functions. Understanding the patterns of brain functional connectivity in patients with narcolepsy in this age group is crucial for elucidating the neurobiological mechanisms of the disease and developing targeted interventions. This study aimed to utilize resting-state fNIRS (rs-fNIRS) to evaluate prefrontal cortex connectivity patterns and network topology in narcolepsy patients compared to matched healthy controls. We hypothesized that the changes in functional connectivity of the prefrontal lobe in the resting state of patients with narcolepsy are closely related to their neurobehavioral characteristics.

## 2 Materials and methods

### 2.1 Participants

**Figure 1:**
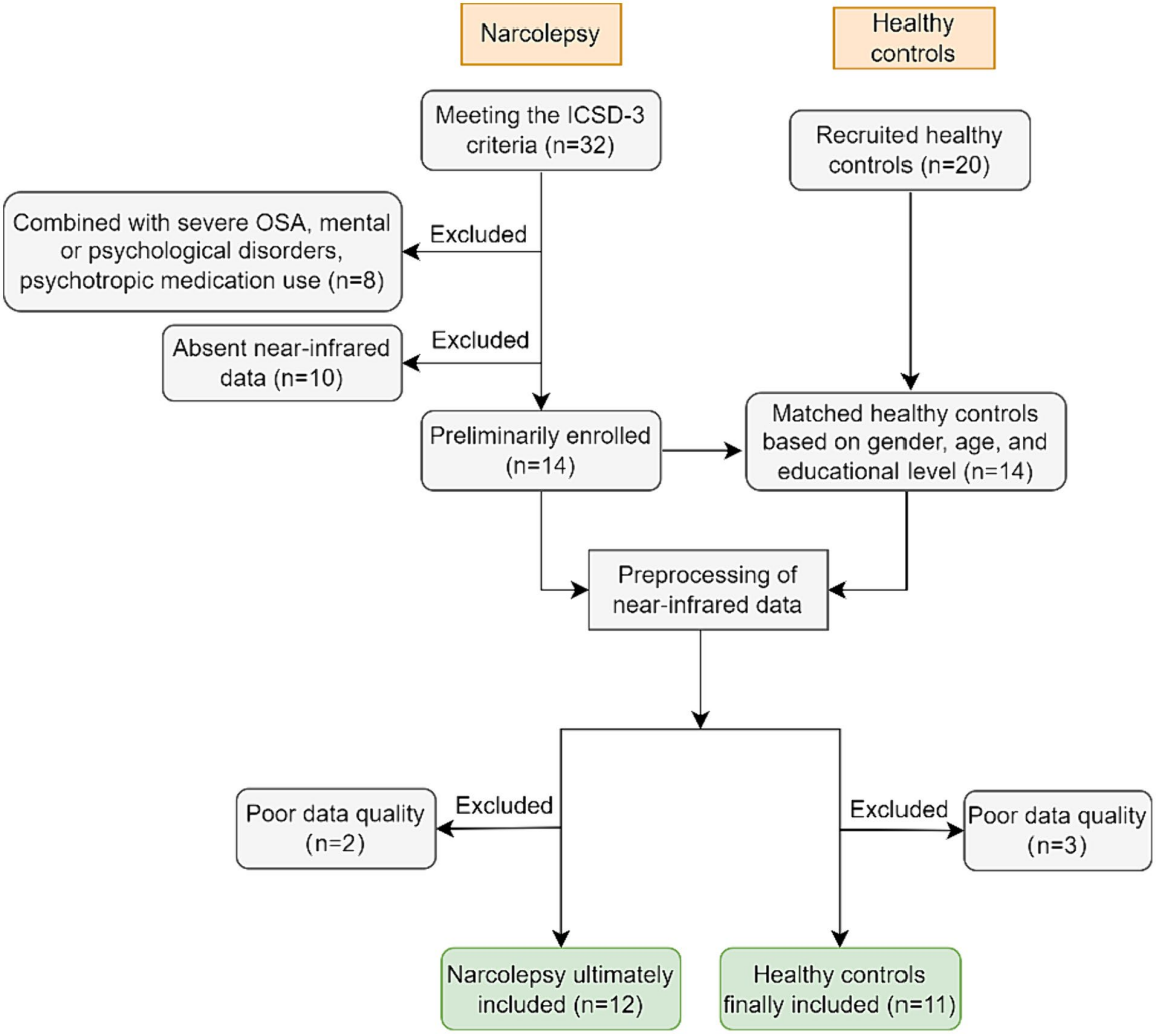
Data acquisition and filtration process diagram.

The study recruited inpatients from the Department of Sleep Medicine at the People’s Hospital of Guangxi Zhuang Autonomous Region from October 2021 to August 2023. Initially, 32 patients who met the ICSD-3 (International Classification of Sleep Disorders, Third Edition, 2014) criteria for narcolepsy were enrolled. Of these, 8 were excluded due to the presence of severe obstructive sleep apnea (OSA), psychiatric or psychological illnesses, or the use of psychotropic drugs. And additional 10 patients were excluded due to missing near-infrared spectroscopy (NIRS) data. Following the preprocessing of NIRS data for the remaining 14 patients, 2 were further excluded due to poor data quality (details in Section 3.1), Consequently, the final cohort consisted of 12 patients (6 males, 6 females; mean age 18.75 ± 3.84). From the initially recruited 20 healthy control subjects, 14 were matched to the narcolepsy patients by gender, age, and educational level. After preprocessing of the near-infrared spectroscopy (NIRS) data, 2 subjects were excluded due to substandard data quality, resulting in a final group of 11 healthy controls (3 males, 8 females; mean age 20.45 ± 4.18) (details in Figure 1).

Narcolepsy patients met the ICSD-3 diagnostic criteria based on clinical assessment and overnight polysomnography. Exclusion criteria for these patients included other sleep disorders, significant neurological or psychiatric comorbidities, history of head injury or loss of consciousness, and use of medications affecting sleep or brain activity. Diagnosis confirmation involved reviewing clinical histories, sleep study results, and CSF hypocretin-1 levels. The diagnostic workup also included neuropsychological assessments, sleep questionnaires, HLA-DQB1 typing, blood tests, multiple sleep latency tests, and CSF analysis, with hypocretin-1 levels measured at Jinyu Medical Biology Laboratory.

Healthy controls were free from sleep disorders, neurological or psychiatric conditions, chronic medical conditions, and medications affecting sleep or brain function. Exclusion also applied to those with abnormal sleep questionnaire findings. All study procedures adhered to ethical standards set by the local ethics committee, and written informed consent was obtained from each participant.

### 2.2 Polysomnography and MSLT

All participants completed evaluations using the Epworth Sleepiness Scale, Self-rating Anxiety Scale, and Self-rating Depression Scale. They were instructed to abstain from smoking, caffeine, and alcohol for at least 24 h prior to participation. The narcolepsy patients then underwent overnight polysomnography followed by a Multiple Sleep Latency Test (MSLT) the next day adhering to American Academy of Sleep Medicine guidelines. During the MSLT, patients had scheduled nap opportunities every 2 h after morning awakening. Nap trials ended after 20 min if no sleep occurred, otherwise sleep latency was defined as the time from lights out to the first epoch of any stage of sleep. Trials continued for at least 15 min after sleep onset to assess for rapid eye movement (REM) sleep. If REM sleep occurred, the REM latency was documented. The mean sleep latency across the 5 nap opportunities (MSLT-SL) and number of REM sleep onsets (SOREMs) were calculated for each patient. The demo-graphic, clinical, and laboratory characteristics of the 12 participants with narcolepsy are presented in Table 1.

**Table 1:** Clinical and laboratory data of patients.

ID	Age (yr)	Sex	Disease duration (years)	HLA-DQB1 gene	CSF Hcrt (pg/mL)	MSLT-SL	MSLT-SOREMPs	MSLT-SOREMs
1	17	M	6	03:02, 06:02	15.4	1.2	2	3
2	16	F	5	None	322.71	6.4	1.7	3
3	18	F	3	None	63.12	4.1	3.7	5
4	15	M	0.5	04:01, 05:02	223.89	6.3	7	2
5	18	M	3	None	219.92	3.8	11	2
6	17	F	2	05:01, 06:02	126.18	3.8	3	4
7	20	F	11	None	27.66	3.8	8.8	5
8	26	F	10	05:02, 06:02	36.05	5.3	7.2	3
9	17	M	7	03:03, 06:02	11.83	0.8	0	4
10	16	F	2	None	–	0.9	2.4	4
11	18	M	3	None	14.49	3	4.5	3

Yr, years; F, female; M, male; HLA-DQB1 Gene: Human Leukocyte Antigen DQB1 Gene. hcrt, hypocretin-1 level in CSF (pathological levels < 110 pg/mL); MSLT-SL, Multiple Sleep Latency Test—Sleep Latency; SOREMPs, Sleep Onset REM Sleep Periods (Average Sleep Latency to SOREM); SOREMs, number of REM sleep onsets.

### 2.3 fNIRS acquisition and processing

In the current study, the acquisition and preprocessing of fNIRS data were performed using the same methodologies as described in our previous work (Mingming et al., 2024). Briefly, a 37 multichannel fNIRS instrument (BS-3000, Wuhan Znion Technology Co., Ltd., Wuhan, China) was employed to monitor the hemodynamic responses in the prefrontal cortex during a 3-min resting state. The channel placement and normalization procedures, along with the conversion of the channels to MNI space and ROI identification, adhered to the protocols previously established (see Figure 1 in our previous works, Mingming et al., 2024 for spatial distribution of fNIRS channels).

## 3 Data analysis

### 3.1 Data pre-processing

Data pre-processing was conducted using the Homer2 MATLAB toolbox, following a sequence of steps that included conversion to optical density, motion artifact correction, band-pass filtering, and calculation of hemoglobin concentration changes using the modified Beer–Lambert Law. The initial 15 s of data were discarded to mitigate the effects of instability, with the subsequent 165 s being baseline-corrected and used for functional connectivity analysis. Channels exhibiting a coefficient of variation (CV) greater than 35% were deemed unreliable and excluded from further analysis. For a detailed description of the pre-processing steps, please refer to our previous study (Mingming et al., 2024).

Ultimately, datasets from 2 narcolepsy patients and 3 healthy controls were excluded from the analysis due to excessive motion artifacts or other criteria that rendered their data unreliable. The flowchart depicting the data exclusion process is illustrated in Figure 1.

### 3.2 Resting-state functional connectivity

**Figure 2:**
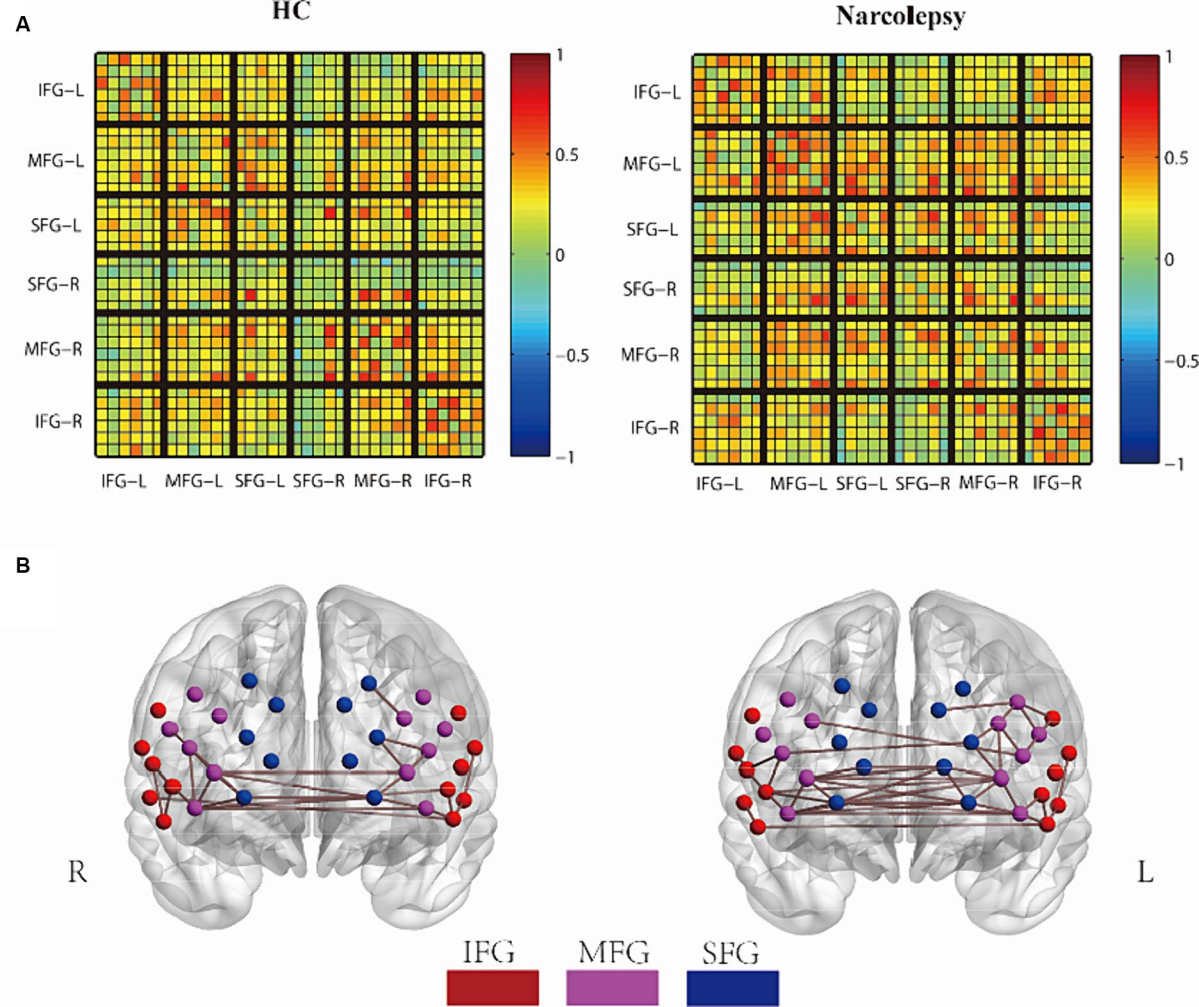
Group comparison of FC networks. **(A)** The mean FC matrix for the HC group (left) and the narcolepsy group (right), Each small grid represents a channel. **(B)** The average 3D network connectivity graph for the HC group (left) and the narcolepsy group (right), with a network threshold of 0.5. In the mean FC matrix, warm colors and cool colors represent positive and negative correlations, respectively. In the 3D network connectivity graph, red points indicate the Superior Frontal Gyrus (SFG), purple points indicate the Middle Frontal Gyrus (MFG), and blue points indicate the Inferior Frontal Gyrus (IFG).

In this study, we constructed resting-state functional connectivity (RSFC) based on oxygenated hemoglobin (HbO2) signals, with all analyses carried out using the NIRS-KIT toolkit (Hou et al., 2021). We calculated the average functional connectivity (FC) matrices for both patient and healthy control groups, with a specific emphasis on intra-hemispheric and inter-hemispheric connectivities based on predefined regions of interest (ROIs). These ROIs included bilateral inferior frontal gyrus (IFG), middle frontal gyrus (MFG), and superior frontal gyrus (SFG), as shown in Figure 1. of our earlier work (Mingming et al., 2024). Connectivity was categorized into four types: intra-hemispheric within ROIs (S1), intra-hemispheric between different ROIs (S2), inter-hemispheric between sym-metrical ROIs (L1), and inter-hemispheric between asymmetrical ROIs (L2), as depicted in Figure 2B of our earlier work (Mingming et al., 2024). When constructing different threshold-based connectivity matrices using NIRS-KIT, we noted that network connections were almost nonexistent at a threshold of 0.55, hence we focused our analysis on a threshold of 0.5.

To portray the topological features of the brain network, we employed graph theory analysis, concentrating on metrics such as degree centrality, betweenness centrality, global network efficiency, local network efficiency, node clustering coefficient, node efficiency, local node efficiency, shortest path length, and small-world characteristics. These metrics allowed us to delve deeper into the network’s capacity for integration and segregation, thus providing a comprehensive understanding of the brain’s complex functional architecture. For a more detailed explanation of these topological measures, we recommend that readers refer to our previous work (Mingming et al., 2024).

### 3.3 Statistical analysis

Statistical analyses were conducted using IBM SPSS version 22 and R version 3.8. Quantitative data that adhere to a normal distribution are presented as the mean ± standard deviation (mean ± SD), with the comparison between two groups conducted using the independent sample t-test. Quantitative data that do not follow a normal distribution are presented as the median with the 25th and 75th percentiles, denoted as M[P25, P75], comparisons between groups are made using the Mann–Whitney U test. Categorical data were analyzed using the chi-square test or Fisher’s exact test. Statistical tests were corrected for multiple comparisons using Benjamini-Hochberg (Benjamini and Hochberg, 1995) to control for false discovery rate (FDR). For inter-network comparisons, Pearson correlation coefficients were calculated for the mean functional connectivity (FC) matrices of the two groups, similar to resting-state fMRI studies (Calhoun and Allen, 2013; Iraji et al., 2016). This involved vectorizing the lower triangles of each FC matrix and correlating these vectors to evaluate the similarity of network organization between groups. Subsequently, partial correlation analysis was conducted between graph-theoretic metrics and clinical characteristics, controlling for gender and age.

## 4 Results

### 4.1 Demographic and clinical score

The final cohort included 12 individuals diagnosed with narcolepsy and 11 healthy control subjects. An analysis of demographic variables revealed no significant differences in gender distribution, age, body mass index (BMI), or years of education between the narcolepsy patients and the control group (see Table 2).

**Table 2:** Demographics and scale scores in narcolepsy vs. healthy controls [ mean±(SD),M(P25,P75) ].

Variables	Narcolepsy	HC	t/U/X^2^	*p-*value
Age (years)	19.50(17.25, 22.75)	17.00(16.50, 18.00)	44.5	0.19^b^
Gender (M/F)	6/6	3/8	–	0.25^a^
BMI	22.31 ± 2.58	22.32 ± 3.00	0.02	0.98
Education (years)	11.92 ± 2.11	13.27 ± 2.69	1.35	0.19
SAS	36.87(35.63, 45.31)	35.00(31.25, 48.75)	36	0.50^b^
SDS	48.47 ± 10.64	42.30 ± 5.66	−1.60	0.13
ESS	13.44 ± 3.84	7.00 ± 2.00	−4.66	0.00

HC, healthy controls; F, female; M, male; SDS, Self-rating Depression Scale; SAS, Self-rating Anxiety Scale; ESS, Epworth Sleepiness Scale. ^a^Fisher’s exact test. ^b^Mann–Whitney U test.

Regarding clinical scale scores, the narcolepsy group had marginally higher scores on the self-rating anxiety scale (SAS) and self-rating depression scale (SDS) compared to the control group; however, these differences did not reach statistical significance, with *p*-values of 0.70 and 0.13, respectively. In contrast, the Epworth Sleepiness Scale (ESS) scores were significantly higher in the narcolepsy patients, with a *p*-value of 0.001, indicating increased daytime sleepiness in this group.

The findings suggest that patients with narcolepsy exhibit higher levels of drowsiness and tend to have higher anxiety and depression scores, although these latter differences were not statistically significant when compared with the control group.

In summary, there were no significant differences in demographic characteristics or most clinical scale scores between the two groups, confirming their comparability for subsequent analyses.

### 4.2 Functional connectivity comparison

The functional connection matrix and 3D connection network of the two groups are shown in Figures 2A,B. In the average FC matrix, the spatial distribution of RSFC in the HC group (left side of Figure 2A) was similar to that of the Narcolepsy group (right side of Figure 2A) (*r* = 0.584, *p* < 0.001). The visual representation of the matrix indicated that overall functional connectivity in patients with narcolepsy was stronger than in healthy controls. When the correlation threshold was set at 0.5, patients with narcolepsy exhibited more connections above this threshold compared to the control group (Figure 2B). However, at a correlation threshold of 0.55, there were almost no significant connections observable in the 3D connectivity network (see Supplementary Figure S1).

In summary, these results suggest that functional connectivity in the prefrontal cortex of patients with narcolepsy is higher than that in matched healthy controls. The increased functional connectivity in the prefrontal cortex of narcolepsy patients may reflect a compensatory mechanism of the brain in response to the disease.

### 4.3 Graph-based analysis

The topological structure of the network was analyzed by using graph theory, and the indexes such as degree center degree, intermediate center degree, global network efficiency, local network efficiency, node clustering coefficient, node efficiency, local node efficiency, shortest path length and small world characteristics were obtained. During the analysis, age and sex were adjusted as covariates to control for potential confounding effects. Both the patients with narcolepsy and the healthy controls exhibited small-world topological networks (λ ≈ 1 and σ > 1) at the chosen Pearson correlation threshold. However, at the specified Pearson correlation threshold, there was no significant difference in small-world network parameters between patients and controls.

Significant differences in certain graph theory indices were observed between the narcolepsy group and the healthy control group, particularly in the average degree of centrality (aDC_ROI_MFG_L), average node efficiency (aNE_ROI_MFG_L), and average node shortest path length (aNLP_ROI_MFG_L) (refer to Table 3). The consistency of these findings across different thresholds underscores their robustness. Notably, significant indicators were primarily confined to the left middle frontal gyrus (MFG_L), suggesting that functional connectivity changes associated with narcolepsy may exhibit lateralization (as shown in Table 3).

**Table 3:** Network metrics with inter-group differences.

Variables	Narcolepsy	HC	*t*	*P*-value	*P*_FDR
ROI threshold					
NLP_ROI_MFG_L_4	1.23 ± 0.04	1.28 ± 0.04	2.81	0.010	0.064
NLP_ROI_MFG_L_5	1.19 ± 0.03	1.23 ± 0.04	2.31	0.031	0.191
NLP_ROI_MFG_L_6	1.16 ± 0.03	1.20 ± 0.05	2.23	0.039	0.239
NLP_ROI_MFG_L_7	1.14 ± 0.03	1.18 ± 0.05	2.33	0.032	0.192
Ne_ROI_MFG_L_4	0.82 ± 0.02	0.79 ± 0.02	−2.82	0.010	0.062
Ne_ROI_MFG_L_5	0.84 ± 0.02	0.82 ± 0.02	−2.24	0.036	0.218
Ne_ROI_MFG_L_7	0.88 ± 0.02	0.85 ± 0.03	−2.24	0.038	0.228
Dc_ROI_MFG_L_4	23.58 ± 1.54	21.61 ± 1.99	−2.65	0.016	0.095
Dc_ROI_MFG_L_5	25.07 ± 1.56	23.44 ± 1.89	−2.25	0.036	0.219
Dc_ROI_MFG_L_6	26.42 ± 1.48	24.61 ± 2.26	−2.25	0.038	0.225
Dc_ROI_MFG_L_7	27.44 ± 1.86	25.36 ± 1.48	−2.25	0.036	0.218
ROI average					
aDc_ROI_MFG_L	12.63 ± 0.80	11.70 ± 1.24	−2.15	0.044	0.303
aNe_ROI_MFG_L	0.42 ± 0.01	0.41 ± 0.02	−2.27	0.037	0.223
aNLP_ROI_MFG_L	0.60 ± 0.01	0.62 ± 0.03	2.51	0.024	0.141

ROI Thresholds indicate the values for each Region of Interest at different thresholds. The numbers 4, 5, 6, 7 correspond to thresholds of 0.55, 0.6, 0.65, 0.7, respectively. aNe, average nodal efficiency; aNLP, average nodal shortestopath; aDc, average degree centrality.

However, after applying the false discovery rate (FDR) correction, none of the results remained statistically significant. This indicates that a larger sample size may be required to corroborate these preliminary findings.

### 4.4 Brain network and clinical correlations in narcolepsy

**Figure 3:**
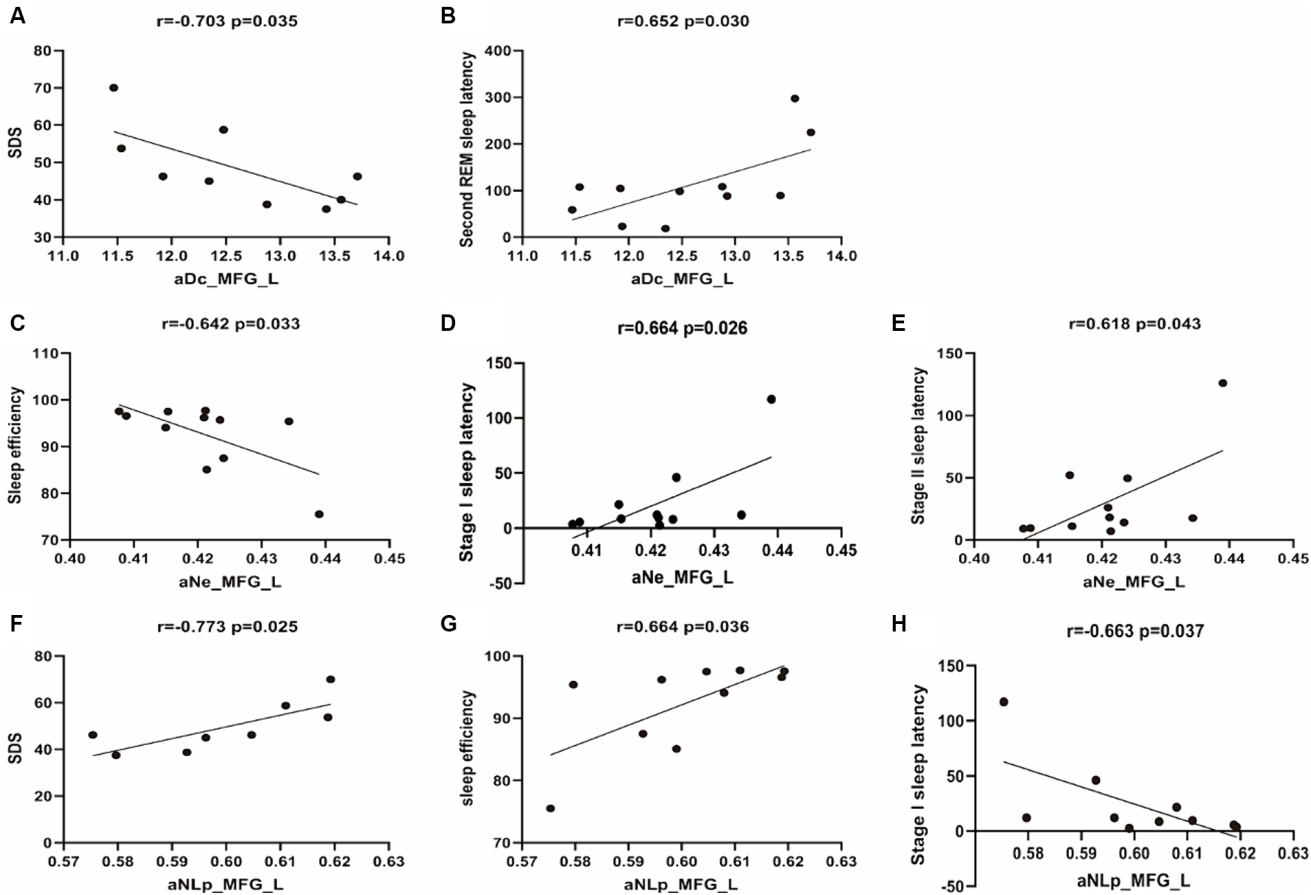
Scatterplots of correlation analyses between network metrics and clinical characteristics. The black line represents the linear fitting trend. SDS, Self-rating Depression Scale; aNe, average nodal efficiency; aNLP, average nodal shortestopath; aDc, average degree centrality.

To evaluate the clinical relevance of altered functional brain networks in narcolepsy, correlation analyses were conducted between fNIRS connectivity metrics and a comprehensive set of data encompassing demographic characteristics, clinical scale scores, Multiple Sleep Latency Test (MSLT) outcomes, and polysomnography results (see Table 4 and Figure 3).

**Table 4:** Correlation analysis of network metrics with clinical indices in the narcolepsy group.

Variables	aDc_MFG_L(r,p)	aNe_MFG_L(r,p)	aNLp_MFG_L(r,p)
Self-rating depression scale	−0.703, 0.035	−0.645, 0.061	−0.773, 0.025
Polysomnography			
Sleep efficiency	−0.590, 0.056	−0.642, 0.033	0.664, 0.036
Second REM sleep latency	0.652*, 0.030	0.446, 0.169	−0.495, 0.146
Stage I sleep latency	0.507, 0.112	0.664*, 0.026	−0.663*, 0.037
Stage II sleep latency	0.446, 0.169	0.618*, 0.043	−0.621, 0.056

r, Pearson correlation coefficients; p, *p*-values. Second REM Sleep Latency refers to the latency period before the second occurrence of REM sleep during the monitoring period.*The statistical results are significant.

The average degree centrality (aDc_MFG_L) measured in the left middle frontal gyrus shows a significant negative correlation with self-rated depression scores (*r* = −0.703, *p* = 0.035), suggesting that lower centrality is associated with higher depression scores. It also shows a significant positive correlation with Second REM Sleep Latency (*r* = 0.652, *p* = 0.030). No significant correlation was observed with sleep onset latency during Stage I or II, or with sleep efficiency (*r* = 0.507, *p* = 0.112; *r* = 0.446, *p* = 0.169; *r* = −0.590, *p* = 0.056, respectively).

The average nodal efficiency in the left middle frontal gyrus (aNe_MFG_L) is negatively correlated with sleep efficiency (*r* = −0.642, *p* = 0.033) and positively correlated with sleep onset latency during Stage I and Stage II (*r* = 0.664, *p* = 0.026; *r* = 0.618, *p* = 0.043). No significant correlation was observed with self-rated depression scores or Second REM Sleep Latency (*r* = −0.645, *p* = 0.061; *r* = 0.446, *p* = 0.169).

The average nodal shortest path length in the left middle frontal gyrus (aNLp_MFG_L) has a significant negative correlation with self-rated depression scores (*r* = −0.773, *p* = 0.025), indicating that higher depression scores are associated with a more efficient or shorter path within the MFG_L network. Additionally, it shows a positive correlation with sleep efficiency (r = 0.664, *p* = 0.036), and a negative correlation with sleep onset latency during Stage I (*r* = −0.663, *p* = 0.037). However, no significant correlation was observed with sleep onset latency during Stage II or Second REM Sleep Latency (*r* = −0.621, *p* = 0.056; *r* = −0.495, *p* = 0.146).

## 5 Discussion

This study utilized resting-state functional near-infrared spectroscopy to examine prefrontal cortex connectivity patterns in narcolepsy patients compared to healthy controls. The results revealed increased overall functional connectivity strength in narcolepsy patients. Graph theory analysis further identified altered network topology metrics localized to the left middle frontal gyrus. These included increased degree centrality, nodal efficiency, and decreased shortest path length.

### 5.1 Increase in RSFC in narcolepsy

Our study reveals an increase in resting-state functional connectivity (RSFC) within the prefrontal cortex of narcolepsy patients when compared to healthy controls. This increase in connectivity strength may indicate a potential compensatory response.

This observation is consistent with previous neuroimaging studies that have documented increased prefrontal activity in narcolepsy patients during both task-related and resting states (Dauvilliers et al., 2003; Thomas, 2005; Fulong et al., 2018; Xiao et al., 2019; Fulong et al., 2020; Ballotta et al., 2021). A study by Yves Dauvilliers et al. on narcolepsy with cataplexy using ^18^F-PET/CT found high metabolism in the executive network of patients in a baseline state while fully awake (Dauvilliers et al., 2010). The prefrontal cortex, as a crucial part of the executive network, is essential for functions such as attention, working memory, and behavioral control (Collette and Van der Linden, 2002; Collette et al., 2005; Moraes et al., 2012; Juvodden et al., 2023). Another study using ^18^F-FDG PET/CT showed similar results of enhanced local metabolism (Dauvilliers et al., 2017), the authors believe that this relative increase in local metabolism can be interpreted as reflecting the motivation and cognitive effort of patients to remain vigilant, as has already been reported in obstructive sleep apnea (Ayalon et al., 2006) and Kleine-Levin syndrome (Dauvilliers et al., 2014). Moreover, previous structural MRI and functional MRI studies have also revealed similar findings of functional enhancement. A study that combined measurements of cortical thickness with assessments of local cortical volume in patients with narcolepsy surprisingly observed an increase in cortical volume and thickness in the dorsolateral prefrontal cortex region, which is hypothesized to reflect a compensatory process that maintains cognitive abilities in sleepy individuals (Schaer et al., 2012). Xiao Fulong and others used functional magnetic resonance imaging to assess the resting-state low-frequency amplitude parameters in patients with narcolepsy. Compared to the healthy control group, patients with narcolepsy had higher fALLF values in the bilateral sensorimotor cortex (SMC) and bilateral middle temporal gyrus (MTG) (Fulong et al., 2018). Another functional magnetic resonance study based on BOLD imaging by Fulong et al. (2020). conducted a network-based statistical analysis and found that narcolepsy patients had a set of connectivity enhancements, including the middle and inferior frontal gyrus, CAU, primary motor cortex (precentral gyrus), auxiliary motor cortex, hippocampus and parahippocampal gyrus, superior gyrus and middle gyrus. These brain regions are related to integration of sensory systems, emotional and cognitive control, and maintenance of stress alertness (Sadaghiani et al., 2010; Spetsieris et al., 2015; Uddin, 2015). The increased nodal topological attributes found in narcolepsy patients near these brain regions reinforce their primary role in maintaining arousal levels, reflecting the patient’s efforts to remain alert/awake. These findings collectively support the view that, in the pathophysiological challenges brought by narcolepsy, the enhancement of functional connectivity may act as a neural adaptive strategy to maintain the efficiency of the executive network, thereby preserving cognitive abilities.

In contrast to these findings, however, a substantial corpus of research reports diminished cortical thickness, reduced glucose metabolism, and lower ALFF values in the prefrontal cortex of narcolepsy patients (Joo et al., 2009; Kim et al., 2009; Dang-Vu, 2013; Wada et al., 2019; Cavaliere et al., 2020; Jeon et al., 2020; Wu et al., 2022). These conflicting results may stem from methodological differences in neuroimaging techniques, including changes in data acquisition, preprocessing, analysis, and even patient status during scanning. In addition, the heterogeneity of ictal narcolepsy symptoms and treatment history among study participants may have contributed to these differences. The increased connectivity observed in our study may also reflect a specific stage or phenotype of ictal sleep disorder, suggesting that the effects of this condition on the prefrontal cortex may not be uniform and may change over time or with disease progression. Further studies using longitudinal designs and standardized imaging protocols are essential to unravel these complexities and to develop a more compelling understanding of how episodic sleep disorders affect brain function.

### 5.2 Brain network topology and clinical correlation

To further elucidate the underlying neurobiological mechanisms in narcolepsy, we employed graph theory analysis to investigate the topology of brain networks. Graph theory provides a powerful mathematical framework to characterize complex network structures and to understand the organizational principles of brain connectivity (Goldenberg and Galván, 2015). Our research findings have revealed significant differences in several indicators of brain network topology. Compared to the control group, individuals with narcolepsy exhibited increased node efficiency (NE) and degree centrality (DC), as well as reduced nodal path length (NPL). In graph theory terms, degree centrality reflects the number of links connected to a node, indicating its importance within the network. Node efficiency represents the efficiency of information transfer between a node and all other nodes in the network. Nodal path length is the average of the shortest paths from the node to all other nodes in the graph, providing insight into how interconnected the network is (Watts and Strogatz, 1998; Rubinov and Sporns, 2010).

The increase in Ne and Dc in the left MFG is partially consistent with the functional connectivity results of the present study, reflecting the possibility of a potential subjective effort to remain alert, similar to the findings of Xiao et al. (2019). In patients with paroxysmal narcolepsy, the degree of centrality (Dc) and node efficiency (Ne) of the left middle frontal gyrus were negatively correlated with depression score and sleep efficiency, and positively correlated with sleep latency. This may indicate that as patients’ depressive symptoms worsen, their brain network activity decreases and their efficiency decreases. High degree centrality may make it difficult for the brain to enter deep sleep, thus reducing sleep efficiency. At the same time, the increase in node efficiency seems to be related to the decrease in sleep efficiency, which may mean that it is difficult for the brain to maintain a stable sleep state. In general, narcolepsy shortens the time it takes to fall asleep (Huang et al., 2018), but in this case, the prolonged sleep latency and increased connectivity and efficiency of the brain network may reflect the normalization of the brain network.

The shorter NPL observed in narcolepsy patients may indicate a shift to a more integrated network configuration, which may be an adaptive response to underlying pathological changes (Farahani et al., 2019). Some studies have pointed out that in patients with paroxysmal narcolepsy, hypothalamic neurons decrease and histamine cells increase, which may be a compensatory adjustment to maintain the neurological function needed for wakefulness, these changes may enhance the network connectivity of the hypothalamus and affect the whole brain network, thus shortening the length of the characteristic path (Thannickal et al., 2003; John et al., 2013; Valko et al., 2013; Kim et al., 2022). The results of our partial correlation analysis showed that the shortest path length was negatively correlated with depression self-assessment and sleep latency, but positively correlated with sleep efficiency. This may mean that a more centralized network structure may be associated with the normalization of sleep latency in patients with paroxysmal narcolepsy, while a more decentralized network structure may be associated with lower levels of depression and higher sleep quality. Given the relationship between the left brain and language and logical thinking, the results that these changes are mainly concentrated in the left hemisphere are particularly intriguing. The lateralization hints observed in our study provide a way for future research to reveal how paroxysmal narcolepsy affects these cognitive domains.

Our research is the first to utilize resting-state brain functional Near-Infrared Spectroscopy (fNIRS) to explore the prefrontal cortex connectivity patterns in adolescents and young adults with narcolepsy. These findings confirm previous studies conducted with MRI and PET/CT, showcasing the innovative use of fNIRS as a non-invasive, cost-effective alternative to traditional neuroimaging techniques, with the potential for broader clinical application. Moreover, our study highlights the potential of altered brain network metrics as biomarkers for the severity of narcolepsy and provides preliminary insights into their correlation with clinical symptoms. It also reveals the complexity of narcolepsy and the necessity for personalized diagnostic and treatment approaches.

While our study has made some progress in utilizing resting-state brain functional Near-Infrared Spectroscopy (fNIRS) to explore the prefrontal cortex connectivity in adolescents and young adults with narcolepsy, there are still some noteworthy limitations. In our study, the number of patients with paroxysmal narcolepsy was limited, which limited our subgroup analysis based on clinical characteristics. The lack of hypothalamic orexin data and polysomnography data in the control group limited the clear relationship between network changes and orexin and sleep-related indexes. Moreover, the inherent limitations of the fNIRS technology due to its relatively lower spatial resolution and limited ability to probe deeper brain regions compared to fMRI may inevitably lead to the oversight of some important information. In addition, our analysis focuses only on the prefrontal cortex, and examining more brain regions will provide a more comprehensive picture of the entire neural network. Finally, because of the cross-sectional nature of our study, future longitudinal studies are necessary to track changes in the network over time and the evolution of the response to treatment.

## 6 Conclusion

Our findings reveal enhanced functional connectivity in the prefrontal cortex, specifically the left middle frontal gyrus, in adolescent and young adult patients with narcolepsy, which may suggest a potential neuroadaptive compensatory mechanism. Altered left frontal cortex functional connectivity and topology may serve as biomarkers of narcolepsy severity. Near-infrared functional brain imaging may provide a promising tool for monitoring this condition.

## Data availability statement

The raw data supporting the conclusions of this article will be made available by the authors, without undue reservation.

## Ethics statement

The studies involving humans were approved by the Ethics Committee of the People’s Hospital of Guangxi Zhuang Autonomous Region. The studies were conducted in accordance with the local legislation and institutional requirements. Written informed consent for participation in this study was provided by the participants’ legal guardians/next of kin.

## Author contributions

CW: Conceptualization, Formal analysis, Methodology, Writing – original draft. MX: Validation, Writing – original draft. SL: Data curation, Investigation, Software, Visualization, Writing – original draft. TB: Data curation, Supervision, Validation, Writing – original draft. WY: Data curation, Validation, Writing – original draft. ZM: Conceptualization, Project administration, Supervision, Writing – review & editing. LY: Investigation, Supervision, Writing – review & editing. QL: Supervision, Writing – review & editing. HW: Methodology, Visualization, Writing – original draft. PF: Validation, Writing – original draft.
