# Supplementary material for: Assessing resting-state brain functional connectivity in adolescents and young adults with narcolepsy using functional near-infrared spectroscopy
Source: Front Hum Neurosci. 2024 Mar 28;18:1373043. doi: 10.3389/fnhum.2024.1373043 (PMC11007108; doi:10.3389/fnhum.2024.1373043)
Supplement: Supplementary file 1 [file Data_Sheet_1.DOCX]

Supplementary Material

# Supplementary Figures


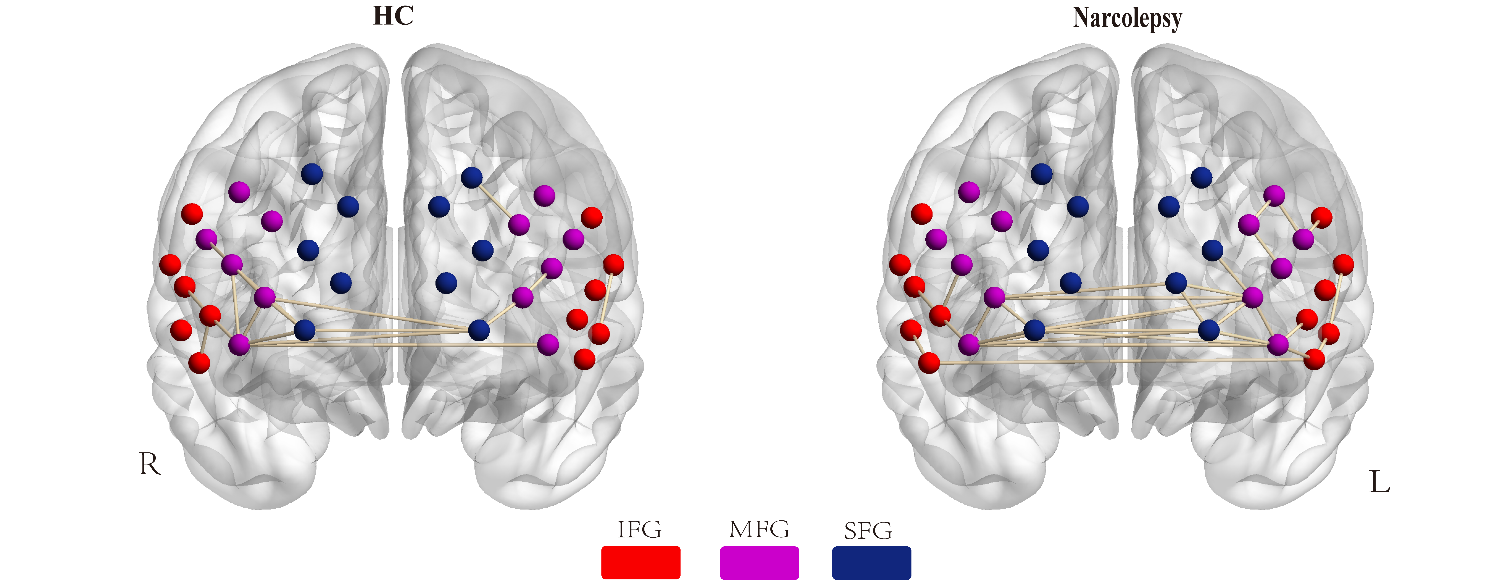


**Supplementary Figure 1.** At a correlation threshold of 0.55, there were almost no significant connections observable in the 3D connectivity network.
